# Overvaluation of shape and weight in adolescents with anorexia nervosa: does shape concern or weight concern matter more for treatment outcome?

**DOI:** 10.1186/s40337-015-0086-7

**Published:** 2015-12-16

**Authors:** Catherine E. Byrne, Andrea E. Kass, Erin C. Accurso, Sarah Fischer, Setareh O’Brien, Alexandria Goodyear, James Lock, Daniel Le Grange

**Affiliations:** Department of Psychology, George Mason University, Fairfax, VA USA; Department of Medicine, The University of Chicago, Chicago, IL USA; Department of Psychiatry, University of California, San Francisco, CA USA; Department of Psychiatry and Behavioral Neuroscience, The University of Chicago Medicine, Chicago, IL USA; Department of Psychiatry and Behavioral Sciences, Stanford University, Palo Alto, CA USA; Department of Psychiatry and Department of Pediatrics, University of California, San Francisco, CA USA

**Keywords:** Overvaluation, Weight, Shape, Anorexia nervosa

## Abstract

**Background:**

Overvaluation of shape and weight is a key diagnostic feature of anorexia nervosa (AN); however, limited research has evaluated the clinical utility of differentiating between weight versus shape concerns. Understanding differences in these constructs may have important implications for AN treatment given the focus on weight regain. This study examined differences in treatment outcome between individuals whose primary concern was weight versus those whose primary concern was shape in a randomized controlled trial of treatment for adolescent AN.

**Methods:**

Data were drawn from a two-site randomized controlled trial that compared family-based treatment and adolescent focused therapy for AN. Chi-square tests and logistic regression analyses were conducted.

**Results:**

Thirty percent of participants presented with primary weight concern (*n* = 36; defined as endorsing higher Eating Disorder Examination (EDE) Weight Concern than Shape Concern subscale scores); 60 % presented with primary shape concern (*n* = 72; defined as endorsing higher EDE Shape Concern than Weight Concern scores). There were no significant differences between the two groups in remission status at the end of treatment. Treatment did not moderate the effect of group status on achieving remission.

**Conclusions:**

Results suggest that treatment outcomes are comparable between adolescents who enter treatment for AN with greater weight concerns and those who enter treatment with greater shape concerns. Therefore, treatment need not be adjusted based on primary weight or primary shape concerns.

## Background

A key diagnostic feature of anorexia nervosa (AN) is an overvaluation of shape and weight [1, 2], with patients tending to evaluate their self-worth in terms of their shape and weight. The Diagnostic and Statistical Manual for Mental Disorders (DSM-5) includes the third diagnostic criterion for AN as “disturbance in the way in which one’s body weight or shape is experienced, undue influence of body weight or shape on self-evaluation, or denial of the seriousness of the current low body weight” [2]. The gold-standard interview assessment of eating disorder pathology, the Eating Disorder Examination (EDE), includes ‘shape concern’ and ‘weight concern’ as two separate subscales [3]. With the exception of one overlapping question, the weight concern subscale and the shape concern subscale on the EDE are comprised of different questions, suggesting the assessment conceptualizes shape and weight concerns as different constructs with distinct latent psychopathology and of separate clinical importance.

Despite the hypothesized clinical importance of distinguishing weight from shape, many factor analytic studies have demonstrated that weight and shape concerns may not represent separate constructs. In fact, two comprehensive literature reviews have demonstrated that factor analyses have generally failed to discriminate between a shape concern factor and weight concern factor on the EDE and EDE-Questionnaire (EDE-Q; the self-report questionnaire of the EDE), suggesting that separating shape and weight concerns may not be a meaningful distinction for many people [4, 5]. However, the research to-date has focused primarily on the validity of the EDE and the EDE-Q as an assessment tool, but the clinical utility of separating these constructs remains a gap in the literature. Additionally, although the psychometric properties of these assessment tools indicate that concerns about weight and shape may overlap, neuroimaging studies indicate that individuals with AN have a neurobiologically distinct response to the estimation of their own body shape [6]. Individuals with AN appear to misperceive their own body shape as larger than the body shape of healthy controls, and also perceive certain body shapes as more desirable than others. These perceptions are associated with unique regions of neural activation, indicating that concerns about ‘shape’ itself are important to patients with AN, and that these misperceptions occur regardless of the actual body weight of the individual [7, 8].

It is necessary for eating disorder treatment research to not only determine the overall efficacy of a particular treatment, but to also identify factors that influence treatment response [9]. Distinguishing predictors that may be modifiable over the course of treatment is of significant importance in successful treatment outcome and the precise delivery of treatment services. Weight (i.e., the number on the scale) is highly relevant to the treatment of both adolescents and adults with AN. Family-based treatment (FBT), which is efficacious for treating adolescents with AN [10], considers weight restoration and re-feeding primary treatment targets, with little discussion topics outside of weight progress in the beginning sessions [11]. Additionally, cognitive-behavioral therapy for AN also prioritizes weight regain during treatment, including “weekly weighing” at every therapy session [12].

Given the focus of weight as a treatment target for patients with AN, it is possible that individuals who endorse heightened valuation of their weight compared to their shape may experience greater difficulty tolerating weight gain, resulting in poorer treatment outcomes. Moreover, given that early weight gain in treatment predicts better outcomes for adolescents with AN [13, 14], understanding factors that may impact treatment engagement and response may inform subpopulations for whom treatment tailoring may be indicated. A better understanding of the clinical importance of shape and weight concerns in this population may help to improve treatment efficacy.

Using data from a two-site randomized controlled trial (RCT) of treatment for adolescents with AN, the purpose of this study was to examine differences in treatment outcome between individuals whose primary concern was weight compared to those whose primary concern was shape. Though reviews of the psychometric properties of the EDE and EDE-Q show that data do not support a distinction between the weight concern and shape concern subscales, the prognostic clinical value of evaluating these as separate concepts has yet to be explored. To our knowledge, this is the first evaluation of weight concern and shape concerns as separate constructs predicting treatment outcome for individuals with AN. Additionally, to our knowledge, this is the first evaluation examining the predictive clinical utility of separating weight concern items and shape concern items during the eating disorder symptom assessment process. We hypothesized that adolescents who endorsed heightened valuation of their weight compared to their shape would experience greater difficulty tolerating weight gain, resulting in poorer treatment outcome.

## Method

### Participants & procedure

Data were drawn from a two-site RCT (Chicago and Stanford) that compared family-based treatment (FBT) and adolescent-focused therapy (AFT) [10]. Adolescents (*N* = 121) meeting DSM-IV criteria for AN, excluding the criterion requiring the absence of at least three menstrual cycles, were randomly assigned to one of the two treatments. Each treatment was delivered for a total of 24 h (twenty-four 1-h sessions for adolescents receiving FBT, thirty-two 45-min sessions for adolescents receiving AFT) over 12 months. In-person study assessments were conducted at baseline and end of treatment (EOT).

For the purposes of the current analyses, participants considered having ‘primary weight concern’ (PWC; *n* = 36, 30 %) endorsed higher baseline EDE Weight Concern subscale scores than Shape Concern subscale scores; participants considered having ‘primary shape concern’ (PSC; *n* = 72, 60 %) endorsed higher baseline EDE Shape Concern subscale scores than Weight Concern subscale scores. Additionally, for the purposes of this study, participants with equal weight and shape concern subscale scores (*n* = 13, 10 %) were excluded from the analyses.

At end of treatment, normal weight was defined as ≥ 95 % of expected body weight for sex, age, and height. Treatment remission was defined as achieving both normal weight and a global EDE score within 1 standard deviation (SD) of published norms (1.59) [15, 16]. Full disclosure of the purpose of the study, the benefits and risks to patients’ participation, and the confidential nature of information obtained during the study is explained to participants per ethics board requirements. The Institutional Review Boards at both sites approved this study, and all adolescents and at least one parent gave informed assent and consent, respectively. Further details of this the study design and main outcome findings are reported elsewhere [10].

### Measures

#### Weight

Weight in pounds was objectively measured by trained assessors at baseline, at every treatment session, and at EOT. At each study assessment, participants were weighed wearing a hospital gown on a balance-beam scale that was regularly recalibrated.

#### Eating Disorder Examination (EDE)

The EDE is a semi-structured interview used to assess eating disorder pathology [17]. The EDE (version 12.0) was administered at baseline and EOT. The EDE was used to assess psychological and behavioral symptoms of an eating disorder, yielding four subscales (Restraint, Eating Concern, Shape Concern, Weight Concern) and a global score, which indicates level of overall eating disorder pathology. Scores range from 0 to 6, with higher scores indicating greater severity of eating disorder pathology. The EDE has demonstrated good reliability and validity and has been utilized in many studies of youth with eating disorders [5].

### Analysis plan

Analyses were conducted using SPSS Version 22.0. Chi-square tests were used to evaluate between-group differences at baseline between PWC and PSC groups on demographic variables and on treatment condition. Treatment outcome at the end of treatment (i.e., achieving remission or achieving normal weight) was compared by PWC or PSC group using chi-square tests. Logistic regression was used to evaluate the interaction between PWC versus PSC and treatment condition on the proportion of participants achieving remission or achieving normal weight at end of treatment. *P*-values less than 0.05 were considered statistically significant.

## Results

Participants were mostly females (89.8 %) with a mean age of 14.47 years (*SD* = 1.6). Participants identified as 75 % non-Hispanic Whites (*n* = 81), 8.3 % Hispanic White (*n* = 9), 11.1 % Asian (*n* = 12), and 5.6 % as Other (*n* = 6). Mean percent expected body weight was 80.6 % (*SD* = 3.58) using Centers for Disease Control and Prevention growth charts. The average duration of illness was 11.9 months (*SD* = 12.0). Mean difference in ‘shape concern’ scores and ‘weight concern’ scores on the EDE among participants was 0.89 (*SD* = 0.31). Adolescents with PWC did not differ significantly from those with PSC on age, gender, racial/ethnic minority status, duration of illness (months), baseline percent expected body weight, baseline EDE global score, treatment dropout status, or prior hospitalization (*p*s > .165) (See Table 1).

**Table 1:** Baseline and demographic variables

	Primary Weight Concern (*n* = 36)	Primary Shape Concern (*n* = 72)	Statistic	*p*-value
	Mean (SD)	Mean (SD)		
Age (in years)	14.17 (1.483)	14.62 (1.626)	*t* (106) = 1.39	.165
Duration Ill (mo)	11.64 (9.04)	12.09 (13.28)	*t* (106) = .184	.855
Percent EBW	80.91 (3.90)	80.43 (3.43)	*t* (106) = −.646	.520
	*n* (%)	*n* (%)		
Female	32 (90.2 %)	65 (88.9 %)	χ^*2*^ (1) = 0.051	.532
White	26 (72.2 %)	55 (76.4 %)	χ^*2*^ (1) = 0.431	.934
Prior Hospitalization	17 (47.2 %)	32 (44.4 %)	χ^*2*^ (1) = 0.075	.472
Treatment Dropout	9 (25 %)	18 (25 %)	χ^*2*^ (1) = 0.589	.745

Key: *mo* months, *EBW* Expected Body Weight

No significant differences were found at baseline between the PWC and PSC groups by treatment condition (χ^2^ = 0.464, *p* = .496). There were no significant differences between the two groups across treatments in the proportion of participants achieving remission (χ^2^ = 0.702, *p* = .482; PWC achieving remission *n* = 12, 33.3 %, PSC achieving remission *n* = 18, 30 %) or normal weight (χ^2^ = 0.204, *p* = .659; PWC achieving normal weight *n* = 13, 42 %, PSC achieving normal weight *n* = 23, 31.1 %). The interaction between the effect of PWC/PSC group status and treatment condition was not associated with achieving remission (Exp(B) = 0.495, *p* = .457) or normal weight (Exp(B) = 1.300, *p* = .775) post treatment.

A secondary analysis was conducted among participants with clinically-significant baseline weight and shape pathology. Specifically, analyses were conducted among the subset of participants (*N* = 26) with an EDE score greater than or equal to 4 on either the weight concern subscale (*n* = 5) or the shape concern subscale (*n* = 21), given that a score of 4 on a subscale is considered clinically significant [18, 19]. Mean difference in EDE ‘shape concern’ and ‘weight concern’ scores among this subset was 1.14. There were no significant differences between these two groups across treatments in the proportion of participants achieving remission (χ^2^ = 1.250, *p* = .264) or normal weight (χ^2^ = 1.544, *p* = .214).

## Discussion

The current study was an exploratory analysis, examining weight concern and shape concern as separate constructs predicting remission from eating disorder treatment among adolescents with AN. Given the importance of overvaluation of shape and weight as a key clinical characteristic of AN, it is of clinical significance to understand the ways in which these concerns may separately influence the outcome of treatment. Results of this study revealed there were no differences in demographics, weight, or eating disorder pathology between adolescents who presented to treatment with primary weight concern versus with primary shape concern, nor was there a significant difference in clinical outcomes (i.e., remission, normal weight) at the end of treatment. Further, the interaction between treatment group did not moderate the effect of primary weight versus shape concerns on achieving remission or normal weight at end of treatment. Thus, results of this study suggest no significant differences between the two constructs on predicting treatment results among adolescents with AN and indicate that individuals with higher weight concerns are as likely as those with higher shape concerns to have good treatment outcome.

Results of the study were contrary to the hypothesis that, given the focus on weight regain as a treatment target for AN, individuals who place a higher value on weight than shape may have less successful treatment outcomes. Although weight (i.e., the specific number on the scale) restoration is often a primary treatment target for adolescents with AN, it is possible that changes in weight are quickly associated with changes in shape, reducing the prognostic utility of these two constructs to differentiate clinical outcomes by the end of treatment. Further research to understand changes over the course of treatment in weight concerns and shape concerns may be beneficial for informing whether temporal differences impact treatment outcome.

Study results also contribute to the assessment literature on weight versus shape concerns regarding the prognostic value of assessing these features as separate constructs. Although the original validation study of the EDE showed that the weight concern and shape concern subscales could not be combined despite being closely associated with one another [20], several factor analytic studies have failed to differentiate between a shape concern factor and weight concern factor on the EDE and EDE-Q [5], which is consistent with our study findings. Thus, results from the present study further inform assessment issues by calling into question whether it is clinically meaningful to assess shape concern and weight concern as separate constructs in terms of its prognostic value for AN treatment outcomes.

There are several limitations of the current study that should be noted. First, this study defined primary weight concern and primary shape concern as any difference between these two subscales, regardless of the magnitude of this distinction, as the limited sample size of adolescents in the study precluded setting a more conservative distinction between the groups. Accordingly, the mean difference of 0.89 (SD = 0.31) was small, which may have limited the capacity to observe differences in outcome between these groups. However, this also suggests that the mean difference between weight and shape concern tends to be low, which is in keeping with a unified weight and shape concern construct. Additionally, the current study examined a treatment-seeking sample of adolescents with AN, and thus study results may not generalize to other eating disorder samples.

## Conclusions

Despite these limitations, this study represents the first to examine whether weight concern and shape concern differentially impact treatment outcome for adolescents with AN. This study makes a clinical contribution in understanding the influence that weight concern and shape concern have as two separate components of AN, and suggests that treatment outcomes are comparable between adolescents who overvalue one or the other. Further, it challenges the clinical relevance of assessing weight concern and shape concern as separate constructs.

## Abbreviations

AFT: adolescent-focused therapy
AN: anorexia nervosa
DSM-5: Diagnostic and Statistical Manual for Mental Disorders
EDE: Eating Disorder Examination
EOT: end of treatment
PSC: primary shape concern
PWC: primary weight concern
RCT: Randomized control trial
SD: standard deviation
